# Vitamin B12 Deficiency in Patients Taking Metformin: Pathogenesis and Recommendations

**DOI:** 10.7759/cureus.68550

**Published:** 2024-09-03

**Authors:** Noor ul Huda Ramzan, Khadija Shahjahan, Rubaid A Dhillon, Nimra Tul Ain Khan, Muhammad Bilal Hashmat, Mian Uman Anwer, Dawood Ahmed, Fazila Afzal, Muhammad Mashhood Tahir, Ayesha Muzaffar

**Affiliations:** 1 Medicine, University Medical and Dental College, Faisalabad, PAK; 2 Medicine, Faisalabad Medical University, Faisalabad, PAK; 3 Medicine, Riphah International University, Rawalpindi, PAK; 4 Medicine, Ayub Medical College, Abbottabad, PAK; 5 Medicine, Allied Hospital, Faisalabad, PAK; 6 Emergency, Merit Health, Hattiesburg, USA

**Keywords:** diabetes mellitus, vitamin b12 deficiency, supplementation, screening, vitamin b12 deficiency anemia, neurological outcomes, metformin side effects

## Abstract

Metformin is a cornerstone therapy for type 2 diabetes mellitus due to its glucose-lowering efficacy and additional benefits such as reducing cardiovascular mortality. However, accumulating evidence suggests an association between long-term metformin use and vitamin B12 deficiency, which can lead to serious clinical consequences. This review aims to synthesize current knowledge on the pathogenesis, prevalence, clinical implications, and management of metformin-induced vitamin B12 deficiency. Given the significant clinical implications, it is crucial to monitor and manage vitamin B12 levels in patients using metformin. This review emphasizes the importance of early detection and supplementation to prevent adverse outcomes. By analyzing the current evidence, the review aims to inform healthcare professionals about best practices for managing vitamin B12 deficiency in patients on metformin, offering insights to guide future clinical practices and research directions.

## Introduction and background

According to the World Health Organization (WHO), 422 million people, or just over 5% of the world population, suffer from diabetes [1]. Diabetes is directly responsible for 1.5 million deaths yearly, making it one of the leading causes of mortality worldwide. Due to the steady increase in prevalence, the WHO estimates that by 2040, over half a billion people will have diabetes. In the United States, of the 37.3 million diabetic patients, almost 20 million are prescribed metformin, making it the third most commonly prescribed medication in the country [2,3]. Globally, metformin users crossed 120 million in 2012 [4].

Metformin belongs to a class of drugs called biguanides and was first introduced in Europe in 1958 [5]. It decreases glucose production in the liver, increases insulin sensitivity, and improves glucose uptake in muscle cells [6,7]. The net result of these actions is a reduction in blood glucose levels. It achieves this by activating AMP-activated protein kinase, an enzyme that regulates energy metabolism [6]. This activation results in the suppression of gluconeogenesis and glycogenolysis in the liver. It also increases glucose uptake in muscle cells through the glucose transporter 4 [7].

Since its inception, metformin has been used as an adjunct to diet and exercise to improve glycemic control in adults with type 2 diabetes mellitus (T2DM). It is also used off-label for the treatment of polycystic ovary syndrome and gestational diabetes mellitus [8,9]. Metformin is generally available in extended-release (ER) forms, taken with the evening meal, and immediate-release forms, taken multiple times daily [10]. The ER form has the advantages of fewer gastrointestinal side effects and increased patient compliance [10]. In addition to its glucose-lowering effects, metformin reduces cardiovascular mortality, all-cause mortality, and cardiovascular events in coronary artery disease patients [11]. Metformin reduces the risk of cardiovascular disease by improving lipid profiles, reducing insulin resistance, reducing inflammation, and decreasing oxidative stress [12]. Some studies have also shown that using metformin reduces the risk of various cancers, such as pancreatic, colon, and hepatocellular cancers; however, the precise mechanisms remain subject to ongoing investigation [13].

Metformin can cause gastrointestinal side effects such as nausea, diarrhea, and abdominal discomfort, which typically diminish within a few days or weeks. These are often alleviated by taking it with food and starting at a low dose before gradually increasing it [14]. Metformin can rarely lead to lactic acidosis, especially in individuals with kidney or liver disease, alcoholism, or heart failure. Therefore, kidney function should be monitored regularly in people taking metformin [14].

Additionally, in recent years, it has emerged that metformin might be a culprit in causing vitamin B12 deficiency in susceptible individuals. Various clinical studies worldwide have studied this association and found statistically and clinically significant results supporting this hypothesis. It is imperative to explore this association further, as vitamin B12 deficiency can lead to irreversible neurological damage and anemia, among other clinical manifestations [15,16]. This article aims to provide a comprehensive overview of the mechanisms by which long-term metformin use may lead to vitamin B12 deficiency, examine the prevalence and clinical consequences, and offer evidence-based guidelines for monitoring and managing affected patients. By consolidating the latest research, this review informs healthcare providers on best practices for mitigating the risk of vitamin B12 deficiency in patients prescribed metformin, thereby enhancing patient care and preventing irreversible complications.

## Review

Overview of vitamin B12

Vitamin B12, primarily found in animal products like meat, fish, poultry, eggs, and dairy, has limited plant-based sources, though fortified cereals, nutritional yeast, and algae are exceptions. B12 is essential for the production of DNA and red blood cells, the proper functioning of the nervous system, along with the metabolism of fatty acids and amino acids [15].

Serum vitamin B12 levels are commonly used as a biomarker for B12 status, with true deficiency defined as concentrations below 148 pmol/L and borderline deficiency below 200 pmol/L. However, the test may be misleading as it measures the total amount of vitamin B12 in the blood, including inactive forms. Holotranscobalamin, transcobalamin-bound vitamin B12, is the active form and can be an early marker for B12 status [17-19]. Other tests, such as homocysteine and methylmalonic acid levels, can provide a more accurate reflection of vitamin B12 status, and an elevation of these substances confirms vitamin B12 deficiency [17,19].

Vitamin B12 deficiency presents signs like nausea, weight loss, glossitis, yellowing of the skin, and loss of appetite, along with megaloblastic anemia presenting as fatigue, pallor, and weakness [16]. Neurologically, vitamin B12 deficiency can lead to subacute combined degeneration of three nervous system tracts: the dorsal columns, lateral corticospinal tracts, and spinocerebellar tracts due to abnormal myelin formation. It can also cause ataxia, numbness, memory loss, and psychiatric symptoms like depression and dementia. Of note, vitamin B12 deficiency induced by metformin use is associated with an increased risk of cognitive decline and depression [20]. These changes are usually permanent due to the irreversible nature of demyelination and nerve degeneration.

Pathogenesis of metformin-induced vitamin B12 deficiency

The pathogenesis of vitamin B12 deficiency in individuals using metformin is not fully understood, but several mechanisms have been proposed [21-24]. Metformin, a biguanide medication, has a hydrophobic tail that embeds into the hydrocarbon core of membranes, while the positively charged end alters the surface charge of the cell membrane. This alteration disrupts the function of divalent cations, such as calcium, which plays crucial roles in various membrane processes [25]. A consequence of metformin's action on membranes is its potential to act as a calcium channel blocker. Calcium plays a pivotal role in numerous cellular processes, including those involved in absorbing and utilizing vitamin B12 [26]. For instance, the adhesion of substances to cell surface membranes, like the delivery of vitamin B12 through transcobalamin II receptors on DNA-synthesizing cells, is calcium-dependent. Metformin's interference with calcium-dependent processes could, therefore, impede the normal uptake and utilization of vitamin B12 by cells. Additionally, metformin's action as a calcium-dependent membrane disruptor might directly affect the uptake of the B12-intrinsic factor complex by distal ileal cell surface receptors [27]. Since the binding of B12 to intrinsic factors and its subsequent uptake by ileal receptors are calcium-dependent processes, any disruption caused by metformin could impede the absorption of the intrinsic factor vitamin B12 complex by enterocytes [28].

Moreover, metformin is thought to reduce the absorption of vitamin B12 by influencing small bowel motility and bacterial overgrowth. Diabetic patients who often take metformin frequently exhibit alterations in small bowel motility and gut microbiome, inducing bacterial overgrowth. This altered gut environment could hinder the normal absorption of vitamin B12 from the diet [27].

There is also evidence to suggest that metformin may lead to vitamin B12 deficiency by reducing the levels of the intrinsic factor itself [29]. Metformin may contribute to vitamin B12 deficiency by interfering with the production of intrinsic factor, a glycoprotein essential for vitamin B12 absorption in the ileum. This interference may be due to metformin's effect on gene expression pathways involved in the synthesis and secretion of intrinsic factors [29]. It has also been suggested that metformin may interfere with the metabolism of vitamin B12 in the liver, leading to increased accumulation in the liver and subsequently decreased levels of the vitamin in the blood [29]. Finally, metformin has been shown to alter bile acid metabolism, which plays a critical role in the enterohepatic circulation of vitamin B12 [29]. Bile acids facilitate the absorption of vitamin B12 in the ileum, where the vitamin B12-intrinsic factor complex is absorbed. Metformin's influence on bile acid metabolism may reduce the reabsorption of bile acids, thereby impairing enterohepatic circulation and reducing the availability of vitamin B12 for absorption [29].

Clinical evidence of metformin-induced vitamin B12 deficiency

Multiple studies in various regions of the world, including the United States, Netherlands, Portugal, Saudi Arabia, Pakistan, Japan, Nigeria, and South Africa, have shown vitamin B12 deficiency due to metformin. The estimated prevalence ranges from 4.3% to as high as 41% in some studies [25-36]. Though the prevalence varies from country to country, the overall trend of a statistically significant difference in vitamin B12 levels still exists.

The first study comparing vitamin B12 levels in metformin users to nonusers was conducted in Sweden in 2004 by Hermann et al. [30]. The retrospective analysis included 84 patients, and it was found that patients prescribed metformin had a cobalamin level of 289 vs. 395 pmol/L in those not prescribed metformin (p < 0.01). De Jager et al. [31] followed this with a randomized controlled trial (RCT) in the Netherlands in 2010. This study was the first RCT comparing vitamin B12 levels in metformin users vs. placebo. The subjects were followed for four years. After the study period, it was found that the risk of vitamin B12 deficiency was 7.2% higher in patients prescribed metformin compared to placebo. In comparison, the risk of borderline levels was 11.2% higher in individuals taking metformin.

Two more studies were published in Europe in 2013 and 2017 in the Netherlands and Portugal, respectively. A cross-sectional study in the Netherlands by Grootkamphuis et al. [32] determined that 14.1% of patients taking metformin were vitamin B12-deficient compared to 4.4% of those not. The retrospective observational study from Portugal showed that vitamin B12 deficiency was present in 24.7% vs. 15.8% (p = 0.017) of individuals prescribed metformin compared to those not taking metformin [37].

Many studies from Asia have highlighted the association between metformin use and vitamin B12 deficiency. The first case-control study by Sato et al. [33] was conducted in Japan in 2013. The study concluded that the prevalence of vitamin B12 deficiency in the metformin users' group was 13% compared to 8% in nonusers. Additionally, 29% of metformin users were borderline-deficient, compared to 13% of nonusers. The study found an inverse relationship between metformin dosage and vitamin B12 levels (p = 0.02). Miyan and Waris [34] conducted a prospective observational trial in Pakistan in 2020, which included 932 participants. Of these, 3.9% of metformin users were vitamin B12-deficient compared to only 2.1% of nonusers. The results were statistically significant.

In Saudi Arabia, multiple studies have investigated this association. The first, by Alharbi et al. [35] in 2018, concluded that 9.4% of patients using metformin were vitamin B12-deficient, compared to 2.2% of nonusers. The odds ratio for vitamin B12 deficiency in those taking metformin was 4.72. The second study, by Al Saeed and Baraja [36] in 2021, included 307 participants and compared vitamin B12 levels in those taking over 1,000 mg of metformin daily to those taking less than 1,000 mg. About 71% of individuals prescribed over 1,000 mg of metformin had borderline vitamin B12 levels, while 4.3% were deficient. In contrast, individuals taking less than 1,000 mg had a prevalence of 58.3% for borderline deficiency and 2.5% for deficiency (p = 0.0230). Almatrafi et al. [38] conducted a cross-sectional study of 206 patients and found that vitamin B12 deficiency was present in 17.5% of the patients; however, they did not find a significant association between the dose and duration of metformin use.

Wong et al. [39] studied this association in institutionalized elderly diabetic patients in 2018. Their retrospective analysis found that 53.2% of diabetic patients prescribed metformin had a vitamin B12 deficiency compared to 31% of diabetics who were not regularly taking metformin. They also found that higher doses (≥1,500 mg/day) and longer durations (greater than four years) were significantly associated with increased risk, with an adjusted odds ratio of 2.72 for doses ≥1,500 mg/day and 3.00 for durations greater than four years. Al-Fawaeir and Al-Odat [40] evaluated the influence of metformin on serum vitamin B12 levels in 155 Jordanian T2DM patients in 2022. The study reported that the mean serum vitamin B12 level was significantly lower in metformin users (268.5 ± 35.8 pg/mL) than in nonusers (389.5 ± 29.8 pg/mL). The prevalence of definite deficiency (<150 pg/mL) was found to be 32% in the metformin group compared to only 9% in the non-metformin group (p < 0.02). The study also found that patients prescribed metformin for over five years had lower average vitamin B12 levels. Most recently, Huynh et al. [41] published a cross-sectional study from Vietnam in 2024 showing that 29 of 156 patients (18.6%) prescribed metformin had a vitamin B12 deficiency. The study also found that a high dose of metformin greater than 1,000 mg/day increased the odds ratio to 5.25.

The first study in an African setting to examine the association of vitamin B12 deficiency with metformin was conducted by Ahmed et al. [42] in South Africa in 2016. In this cross-sectional study, 121 patients were surveyed, and 34 (28.1%) were found to have low vitamin B12 levels. The prevalence of vitamin B12 deficiency increased with the duration (p = 0.015) and cumulative metformin dose (p = 0.009). On average, the vitamin B12-deficient individuals were 62.3 years old compared to 57 years for those with normal vitamin B12 levels (p = 0.012). A novel finding was that individuals of black South African descent had lower odds of being vitamin B12-deficient. This high prevalence is similar to another African study conducted in Nigeria by Owhin et al. [43]. They conducted a prospective case-control study of 200 subjects and observed vitamin B12 deficiency in 41% of patients taking metformin compared to 20% in metformin-naïve patients (p = 0.001). Interestingly, a cross-sectional study by Fakkar et al. [44] in Egypt found no significant association between metformin use and vitamin B12 deficiency. This study included 100 patients and found that vitamin B12 deficiency was present in 4% of metformin users compared to 2% of nonusers; however, the results were not statistically significant.

Reinstatler et al. [45] were the first to investigate the relationship between metformin use and vitamin B12 deficiency in the United States. They conducted a case-control study in 2012 that included 1,621 patients. It was found that vitamin B12 deficiency occurred in 5.8% of diabetic patients using metformin compared to 2.4% of nonusers (p = 0.0026). However, this study is overshadowed by two large RCTs conducted in the United States by Lohmann et al. [46] and Aroda et al. [47] in 2017 and 2016, respectively. Aroda et al. [47] included 2,155 patients and randomized them to receive either metformin or a placebo. They found low and borderline vitamin B12 levels in 19.1% vs. 9.5% (p < 0.01) of patients taking metformin vs. placebo after five years. After 13 years, the difference was 20.3% vs. 15.6% (p = 0.02). The study concluded that there is a correlation between years of metformin usage and vitamin B12 deficiency. Lohmann et al. [46] randomized 492 breast cancer patients to receive metformin or a placebo. At the end of the six-month study period, patients prescribed metformin saw their vitamin B12 levels drop from 390 pmol/L, whereas a similar drop was not seen in those prescribed a placebo.

Hurley-Kim et al. [48] performed a study using the All of Us database of 36,740 participants and concluded that long-term metformin use led to a higher risk of vitamin B12 deficiency, with the risk increasing for every additional year of use. They found that 7.5% of metformin users had a confirmed vitamin B12 deficiency compared to 6.3% of nonusers. They also found that every year of metformin use was associated with a 5% increased risk of developing vitamin B12 deficiency. These studies are summarized in Table 1.

**Table 1:** Summary of studies investigating the association between metformin use and vitamin B12 deficiency

Author	Study design	Location	Year	Number of participants	Summary
Hermann et al. [30]	Retrospective analysis	Sweden	2004	84	Significantly lower levels of cobalamin in patients using metformin
De Jager et al. [31]	Randomized control trial	The Netherlands	2010	390	Higher risk of vitamin B12 deficiency in metformin users after a four-year follow-up
Reinstatler et al. [45]	Case control	United States	2012	1,621	Vitamin B12 deficiency is found at a significantly higher rate in metformin users
Grootkamphuis et al. [32]	Cross-sectional	The Netherlands	2013	298	Higher prevalence of vitamin B12 deficiency in metformin users
Sato et al. [33]	Case control	Japan	2013	108	Statistically significant inverse relation between metformin dosage and vitamin B12 levels
Ahmed et al. [42]	Cross-sectional	South Africa	2016	121	Individuals with a longer duration of therapy and higher cumulative dose of metformin had significantly greater rate of vitamin B12 deficiency
Aroda et al. [47]	Randomized controlled trial	United States	2016	2,155	After five years, vitamin B12 deficiency occurred at a significantly higher rate in patients using metformin
Bello et al. [37]	Retrospective observational	Portugal	2017	1,007	Vitamin B12 deficiency was found at a higher rate in metformin users
Lohmann et al. [46]	Randomized controlled trial	United States	2017	492	In breast cancer patients, a drop in vitamin B12 levels was observed to patients prescribed metformin. No significant difference in vitamin B12 levels was observed in patients prescribed placebo
Alharbi et al. [35]	Retrospective case control	Saudi Arabia	2018	412	Higher percentage (9.4% vs. 2.2%) of Vitamin B12 deficiency in those prescribed metformin vs. those not prescribed metformin
Wong et al. [39]	Retrospective observational	China	2018	1996	53.2% of diabetics prescribed metformin had a vitamin B12 deficiency compared to 31% diabetics not prescribed metformin
Owhin et al. [43]	Prospective case control	Nigeria	2019	200	Vitamin B12 deficiency was found in 41% of the patients taking metformin compared to 20% in metformin naïve patients (p = 0.001)
Miyan and Waris [34]	Prospective observational	Pakistan	2020	932	Higher incidence of vitamin B12 deficiency in patients taking metformin
Al Saeed and Baraja [36]	Observational Cross-sectional	Saudi Arabia	2021	307	Significantly lower vitamin B12 levels in patients taking over 1,000 mg of metformin compared to those taking less than 1,000 mg
Almatrafi et al. [38]	Cross-sectional	Saudi Arabia	2022	206	Vitamin B12 deficiency was presented in 17.5% of patients using metformin. No significant association between the dose and duration of metformin with vitamin B12 deficiency was found
Al-Fawaeir and Al-Odat [40]	Cross-sectional	Jordan	2022	155	Vitamin B12 deficiency was found in 32% of patients on metformin compared to 9% not on metformin
Fakkar et al. [44]	Cross-sectional	Egypt	2022	100	No statistically significant association was found
Hurley-Kim et al. [48]	Retrospective Analysis	United States	2023	36,740	7.5% of metformin users had a confirmed vitamin B12 deficiency compared to 6.3% of nonusers. Metformin use was also associated with a 5% increased likelihood of developing a vitamin B12 deficiency for every year of use
Huynh et al. [41]	Cross-sectional	Vietnam	2024	156	18.6% of patients taking metformin were found to be vitamin B12 deficient

Prevention and treatment of metformin-induced vitamin B12 deficiency

Prophylactic treatment with vitamin B12 is not yet recommended; however, the Medicines and Healthcare Products Regulatory Agency 2022 guidelines suggest periodic monitoring of vitamin B12 in certain individuals prescribed metformin [49]. Based on the current evidence, there is a list of criteria for cost-effective vitamin B12 deficiency screening in metformin-treated patients to identify high-risk individuals who may require vitamin B12 supplementation. These individuals should be screened even if they are asymptomatic for deficiency. This may be useful to prevent the development or worsening of the clinical consequences of vitamin B12 deficiency, particularly anemia and peripheral neuropathy, by allowing for a prompt diagnosis and treatment of vitamin B12 deficiency. The screening criteria for vitamin B12 deficiency are summarized in Table 2.

**Table 2:** Screening criteria outlined for vitamin B12 deficiency MUI: metformin usage index; PPIs: proton pump inhibitors; H2RAs: H2 receptor antagonists

S. no.	Screening criteria for vitamin B12 deficiency
1.	Strong clinical suspicion of deficiency, such as unexplained macrocytic anemia, and neurological symptoms, such as peripheral neuropathy, defined by abnormal monofilament examination, based on findings from the Diabetes Prevention Program/Diabetes Prevention Program Study [31]
2.	In diabetic patients treated with metformin who already have peripheral and/or autonomic neuropathy due to diabetes
3.	Metformin treatment for five years or longer
4.	Elderly individuals aged 65 years and older
5.	High cumulative metformin exposure, indicated by an MUI value exceeding 5, applies to individuals diagnosed with type 2 diabetes who have been treated with metformin for a minimum of six months. The MUI is calculated as the daily metformin dose (in mg) multiplied by its duration (in years) and divided by 1,000 [50]
6.	A metformin dosage of 1,500 mg per day or more, maintained for at least six months, with the greatest risk of vitamin B12 deficiency noted at a daily metformin dose of 2,000 mg or higher
7.	Simultaneous prolonged use (≥12 months) of acid-suppressing medications such as PPIs and H2RAs
8.	Presence of comorbidities associated with increased risk of vitamin B12 deficiency warrants screening based on clinical judgment

Treatment for metformin-induced vitamin B12 deficiency

The treatment for metformin-induced vitamin B12 deficiency is the same as for other causes of vitamin B12 deficiency, which includes dietary modification and supplements. Synthetic vitamin B12 has been widely accessible in the form of cyanocobalamin, available for both oral and injectable administration. More recently, naturally occurring variants such as hydroxycobalamin, methylcobalamin, and adenosylcobalamin have entered the market as supplements for individuals with vitamin B12 deficiency. Although evidence suggests that high-dose oral and parenteral administration of vitamin B12 are equally effective, initial parenteral administration is suggested for symptomatic patients to ensure rapid absorption and compliance [51,52]. A switch to oral therapy is then recommended for the patient's convenience.

As discussed, metformin hinders calcium-mediated absorption of vitamin B12. Studies have demonstrated efficacy in reversing metformin-induced vitamin B12 malabsorption with oral calcium [53]. The study by Bauman et al. [53] reported a decline in total serum vitamin B12 after three months of metformin use. Subsequently, the study observed increased serum vitamin B12 levels after these patients were administered 1.2 g of oral calcium carbonate per day for one month. Patients who take metformin and do not consume milk or milk products or take supplemental calcium should be encouraged to increase their calcium intake. Because vitamin B12 is affordable, easy to administer, safe, and well tolerated, tailoring its use to meet the specific needs of individual patients is generally considered harmless. However, while vitamin B12 supplementation is usually safe, specific populations may face potential risks. For instance, patients with conditions like Leber's disease or polycythemia vera require careful monitoring, as supplementation could potentially exacerbate their conditions [54]. In some individuals, decreasing the dose to less than 1,500 mg/day is associated with protection from vitamin B12 deficiency [55]. Given the excellent safety and effectiveness of metformin as an insulin-sensitizing medication, it is strongly advised to continue it in patients newly diagnosed with vitamin B12 deficiency, as repletion is adequate to prevent further harm. However, it is advisable to regularly monitor vitamin B12 levels in patients treated with metformin, as they may continue to be at higher risk for deficiency despite adequate supplementation.

Strengths and limitations

The strength of this paper is the diversity in the location of the studies included in the review. At the same time, the major limitation of the article is that it focuses on a single adverse effect: vitamin B12 deficiency. Case reports suggest metformin might also cause hemolytic anemia or nightmares and abnormal dreams [54,56]. These other rare adverse effects were not explored. Another limitation is that only one database, PubMed, was used.

## Conclusions

Despite metformin's high efficacy in lowering blood sugar levels, clinical evidence indicates that metformin use can lead to vitamin B12 deficiency. Although the exact mechanism of this association is still unclear, this paper seeks to consolidate and affirm the growing body of evidence concerning metformin-induced vitamin B12 deficiency. Our literature search found that vitamin B12 deficiency ranges from 4.3% to 41% in patients using metformin. Despite regional variations, a clinically and statistically significant difference in vitamin B12 levels between metformin users and nonusers was evident across the studies. Only one small study from Egypt contradicted this trend.

The clinical implications of this deficiency are significant, as unaddressed, prolonged vitamin B12 deficiency can lead to anemia and irreversible neurological damage, among other adverse effects. Prevention and treatment of vitamin B12 deficiency are simple and effective; therefore, clinicians should periodically monitor vitamin B12 levels in patients prescribed metformin. Prompt administration of exogenous vitamin B12 is recommended when a deficiency is found; however, treatment with metformin can continue as needed. Finally, more research is required to investigate potential confounders and effect modifiers. As diabetes is prevalent worldwide, regional factors such as diet and environment that might alter this association must be investigated. Additionally, more research is needed to determine whether specific subgroups, such as race or gender, are more prone to developing vitamin B12 deficiency. This will help tailor guidelines and lead to better patient outcomes for those who are most susceptible.
